# Interleukin-33 serum levels in postmenopausal women with osteoporosis

**DOI:** 10.1038/s41598-019-40212-6

**Published:** 2019-03-07

**Authors:** Lia Ginaldi, Massimo De Martinis, Salvatore Saitta, Maria Maddalena Sirufo, Carmen Mannucci, Marco Casciaro, Fedra Ciccarelli, Sebastiano Gangemi

**Affiliations:** 1 School of Allergy and Clinical Immunology, Department of Life, Health & Environmental Sciences, University of L’Aquila and Allergy and Clinical Immunology Unit “Mazzini” Hospital AUSL4, Teramo, Italy; 2 School and Division of Allergy and Clinical Immunology, Department of Experimental Medicine, University of Messina, Messina, Italy; 3 Department of Biomedical and Dental Sciences and Morphofunctional Imaging, University of Messina, Messina, Italy

## Abstract

There are many cytokines involved in the pathogenesis of osteoporosis. So far IL-33 involvement in osteoporotic patients has not yet been studied. IL-33 is a pro-inflammatory cytokine which mediates several immune functions; its involvement in a wide range of diseases, such as atopic dermatitis, asthma, and rheumatoid arthritis, is now emerging. In view of the crucial role of this cytokine in inflammation and bone remodeling, we measured IL-33 levels in the serum of postmenopausal women with osteoporosis. In 50 postmenopausal osteoporotic patients and 28 healthy postmenopausal control women, serum IL-33 levels were measured by enzyme linked immunosorbent assay. In both patients and controls the bone mineral density (BMD) was measured by double-energy X-ray absorptiometry (DXA). Vitamin D, calcium, alkaline phosphatase (ALP), parathyroid hormone (PTH) serum levels, as well as bone turnover markers, such as C-terminal telopeptide of type 1 collagen (CTX) and N-terminal propeptide of type 1 procollagen (P1NP) were also evaluated. In postmenopausal osteoporotic women IL-33 levels were significantly lower compared to healthy controls (3.53 ± 2.45 vs. 13.72 ± 5.39 pg/ml; P = 0.009) and positively correlated respectively with serum PTH (rho = 0.314; P = 0.026) and P1NP (rho = 0.373; P = 0.011) levels, while a statistically significant inverse correlation was observed between serum IL-33 and CTX levels (rho = −0.455; P = 0.002). Our results thus suggest that IL-33 represents an important bone-protecting cytokine which may be of therapeutic benefit in treating bone resorption.

## Introduction

lnterleukin-33 (IL-33), a 30-kDa protein, is a cytokine expressed by mainly stromal cells and upregulated following pro-inflammatory stimulation. Originally described as an inducer of Th2 cytokine production^1^, IL-33 can function as a traditional cytokine, as an alarmin and as a nuclear factor controlling gene transcription. It exerts its biological effects by interacting with the receptors ST2 (IL-1RL1) and IL-1 receptor accessory protein (IL-1RAcP); both these receptors are largely expressed, especially by innate immune and T helper 2 (Th2) cells. More recently, its receptors were also identified on Th1 lymphocytes, regulatory T cells (Treg) and natural killer cells (NK)^2^. IL-33 can promote the pathogenesis of Th2-related diseases like asthma, atopic dermatitis and anaphylaxis. Now, evidence is accumulating that IL-33 plays a key role in the regulation of adaptive and innate immunity, inflammatory processes and responses to environmental stresses, acting as an alarmin^3^.

IL-33 is therefore a multifunctional immunomodulatory cytokine whose pleiotropic nature is reflected in its recently discovered contrasting roles in a wide range of diseases^4,5^. Serum IL-33 level in asthma significantly increases with the development of the disease^6^. In patients with rheumatoid arthritis IL-33 is higher compared to controls and it positively correlates to bone erosions^7,8^. However, IL-33 has also shown several preventing effects in cardiovascular disorders such as atherosclerosis, obesity, type 2 diabetes and cardiac remodeling. Thus, the properties of IL-33 are either pro- or anti-inflammatory, depending on the clinical condition^9^.

Osteoporosis is a systemic disease of the skeleton, whose central characteristics are loss of bone mass, bone mineral density (BMD) decline and disruption of bone microarchitecture; the main consequence is an augmented skeleton fragility, exposing patients to augmented risk of fractures. Bone is subjected to continuous renewal by bone remodeling, during which bone resorption is followed by bone formation^10^. Osteoporosis occurs when the balance between bone resorption, carried out by osteoclasts, and bone formation, mediated by osteoblasts, is disrupted. Estrogen deficiency in postmenopausal women induces bone resorption^11^. Recent discoveries of osteoimmunology, an interdisciplinary investigation field that focuses on the connections between bone and immune system, have definitively shown that bone remodeling is under strict immunological control^12^. The activity of bone cells is modulated by cytokines. Thus, it is of vital clinical importance to find specific cytokines involved in the regulation of osteoblast and osteoclast activity because they could embody suitable targets for the treatment of osteoporosis.

IL-33 is the most lately identified member of the IL-1 cytokine family; two other members of which, namely IL-1 and IL-18, have well-characterized actions on bone cells^10^. IL-1 itself is a stimulator of osteoclast activity and prompts osteoblast production of cytokine receptor activator of NF-kB ligand (RANKL), the main positive controller of osteoclastogenesis. By contrast, IL-18, produced by osteoblasts and macrophages, inhibits osteoclast formation while has a mitogenic effect on osteoblasts^13^.

Although it seems confirmed a role of IL-33 in the pathogenesis of many inflammatory diseases, the same cannot be said for osteoporosis, where IL-33 action is still debated. Most of the information obtained so far are derived from *in vitro* studies and animal models, so their applicability to humans is not yet clear. Moreover, such studies show opposite roles of IL-33 in bone remodeling. Shulze *et al*. demonstrated that IL-33 directly stopped osteoclast formation from bone marrow precursor cells^14^ and Zaiss *et al*. showed that this cytokine shifted the equilibrium from osteoclast to alternatively activated macrophage differentiation *in vitro*^15^. However, IL-33 also reduced osteoprotegerin (OPG) expression by osteoblasts and increased the production of osteoclastogenic factors, inducing bone resorption in inflammatory conditions^16^.

Although some reports suggest that IL-33 stimulates matrix mineralization *in vitro*^17^, others indicate no influence on bone matrix formation^18^. Notwithstanding IL-33 participates in Th2-mediated processes^9^, thus stimulating osteoblast maturation and decreasing osteoclastogenesis^19^, Mun *et al*. found out that lL-33 directly promoted osteoclast differentiation from human monocyte precursors, thus inducing bone resorption indipendently by RANKL pathway^20^. On the contrary, Kiyomiya *et al*. demonstrated that IL-33 inhibited RANKL-dependent osteoclast formation^21^. Similarly, Zhu *et al*. showed that IL-33 had a suppressive effect on osteoclast differentiation through the inactivation of nuclear factor of activated T-cell cytoplasmic 1(NFATc1), the key regulator for RANKL-induced osteoclast formation^22^, and Velickovic *et al*. demonstrated that mice deficient in the IL-33 receptor displayed increased osteoclast formation and low bone mass^23^. Therefore, due to the pleiotropic action of IL-33, its overall effect on bone homeostasis is difficult to investigate and this could explain the controversial results present in the literature.

Till now, the involvement of lL-33 has been shown in allergic, autoimmune and infectious diseases, as well as in atherosclerosis, diabetes and cancer, however its role in patients with osteoporosis has not yet been studied. Moreover, as preclinical scientific investigations continue to reveal a role of IL-33 in bone remodeling, there is a need for preliminary clinical data. To the best of our knowledge, there have not been so far published clinical studies on IL-33 serum levels in osteoporosis. Here we therefore investigated the serum IL-33 levels in post-menopausal osteoporotic patients and their association with clinical and laboratory findings.

## Methods

### Patients

Fifty postmenopausal osteoporotic women (mean age 65.42 ± sd 9.59 years) and 28 age-matched (62.07 ± 8.34) healthy postmenopausal females were enrolled. According to the Helsinki Declaration^24^, after approval by the local ethics committee (Internal Review Board University of L’ Aquila ex “Academic Ethics Committee” D.R. n. 206/2013 modified D.R. n. 46/2 017), and after obtaining written informed consent from each participant, BMD was assessed at hip and lumbar spine by double energy X-ray absorptiometry (DXA) (Hologic QDR 4500 W machine) in all the women who took part in the study. The BMD was expressed as T-score, which is the difference in standard deviations between the mean value of BMD in the healthy reference population and the BMD value of the examined subject. The diagnosis of osteoporosis was formulated according to the current criteria established by the World Health Organization^25^. T-score values lower than −2.5 define osteoporosis, between −1.5 and −2.5 define osteopenia and higher than −1.5 indicates a normal BMD.

Careful physical examination, accurate clinical history, measurement of anthropometric parameters such as weight, height and body mass index, and laboratory and instrumental analysis (spine radiography and vertebral morphometry to highlight any asymptomatic osteoporotic fractures) were performed in each subject. Among laboratory data, serum calcium levels, vitamin D (1α,25-dihydroxyvitamin D_3_), parathyroid hormone (PTH), and alkaline phosphatase (ALP) were assessed. In addition, in patients and controls, measurements of bone turnover markers were also performed. In particular, serum levels of both the resorption marker C-terminal telopeptide of type 1 collagen (CTX) and the osteoformation marker N-terminal propeptide of type 1 procollagen (P1NP) were evaluated, to indirectly assess osteoclast and osteoblast activity, respectively. Neither patients nor controls were taking drugs influencing bone turnover, and none of the studied women was affected by diseases inducing secondary osteoporosis^17^. All clinical and anthropometric data collected from both osteoporotic and healthy controls are shown in Table 1.

**Table 1:** Clinical, laboratory and anthropometric findings (means ± standard deviations) of patients and controls.

	Patients (n = 50)	Controls (n = 28)
Age (years)	65.42 ± 9.59	62.07 ± 8.34
BMI (kg/m2)	27.31 ± 4.29	28.63 ± 4.84
BMD (T-score)*	−3.09 ± 0.66	−0.23 ± 1.13
PTH (pg/mL)	64.93 ± 22.69	67.97 ± 16.26
ALP (U/L)	123.26 ± 62.36	103.57 ± 25.76
CTX (pg/mL)*	429.01 ± 273.58	290.93 ± 180.57
P1NP (ng/ml)	21.10 ± 11.82	24.37 ± 14.35
1,25(OH)_2_-D_3_(pg/mL)	19.12 ± 8.98	22.37 ± 5.89
Ca^++^(mg/dl)	9.16 ± 0.77	7.14 ± 3.58
Fractured subjects (number)*	14	0

Legend: Body mass index (BMI); Bone mineral density (BMD); Parathyroid hormone (PTH); Alkaline phosphatase (ALP); C-terminal telopeptide of type 1 collagen (CTX); N-terminal propeptide of type 1 procollagen (P1NP); 1α,25-dihydroxyvitamin-D_3_ (1,2 S(OH)_2_-D_3_); Serum calcium (Ca^++^).

*Statistically significant difference between patients and controls (p < 0.01).

### IL-33 dosage

Measurements of serum IL-33 were carried out both in women with osteoporosis and in healthy controls. Blood samples, taken by venipuncture, were centrifuged at 3000 rpm for 5 min and the sera obtained were frozen and stored at −20 °C until processing.

The levels of IL-33 protein were assessed by using a sandwich ELISA kit, according to the standard procedure recommended by the manufacturer (USCN LIFE SCIENCE Houston, TX, USA).

Using a microspectrophotometer mod. 340 ATTC (SLT Lab. Instruments Salzburg, Austria), the absorbance was measured at 450 nm and the values obtained were expressed as pg/ml.

The minimum detectable level of IL-33, as reported by the manufacturer of the assay kit, was lower than 5.3 pg/ml. We have also established the lower limit of detection (LLD), according to the following procedure, as suggested by the manufacturer: (mean negative control optical density) + 2 * (Stde v of negative control optical density). Our LLD was 1.9 pg/ml. We also performed a standard curve starting from 7.8 pg/ml to more accurately detect the IL-33 concentrations in the tested serum samples.

### Statistical analysis

We expressed our results as medians ± SEM. The Mann-Whitney test was used to analyze the differences between the two unpaired groups. Correlation between two variables was evaluated with Spearman’s rho. All statistical analyses were performed using SPSS for Windows (version 17.0). P values < 0.05 were established as statistically significant.

## Results

**Figure 1:**
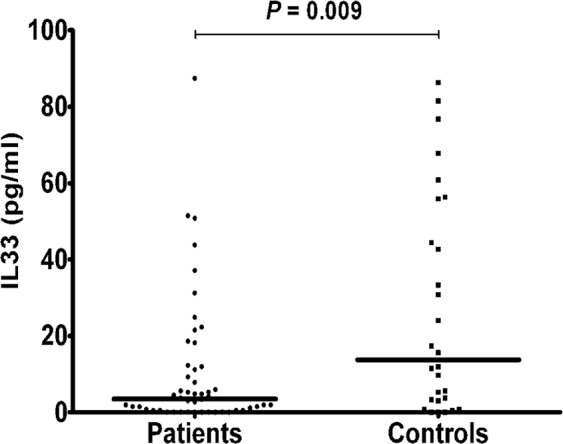
IL-33 serum levels in all patients and controls; lines represent medians.

IL-33 serum levels were statistically lower in osteoporotic patients than controls (3.53 ± 2.45 vs. 13.72 ± 5.39 pg/ml; P = 0.009)(Fig. 1).

**Figure 2:**
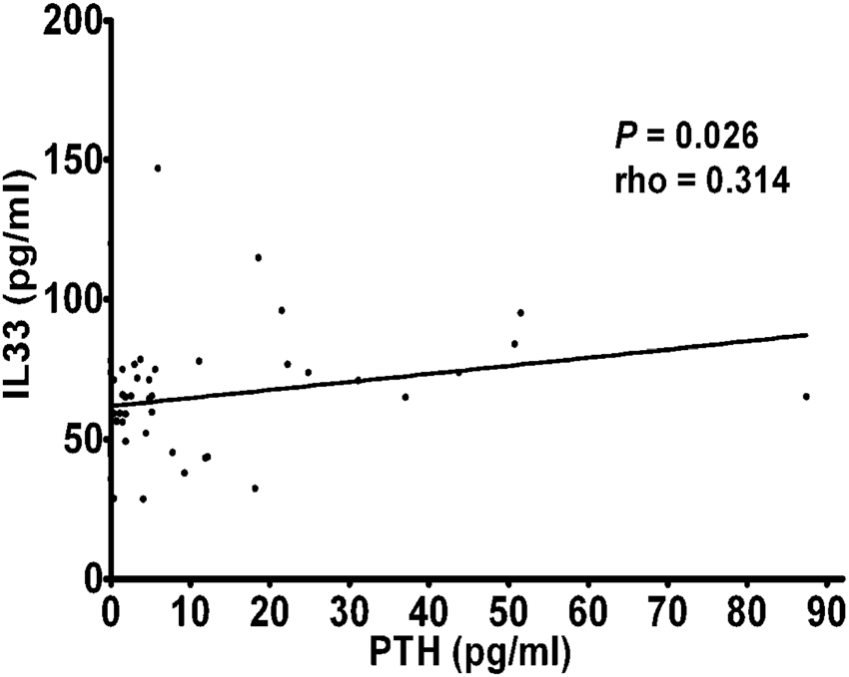
Correlation between IL-33 serum levels and parathyroid hormone (PTH) in osteoporotic patients.

**Figure 3:**
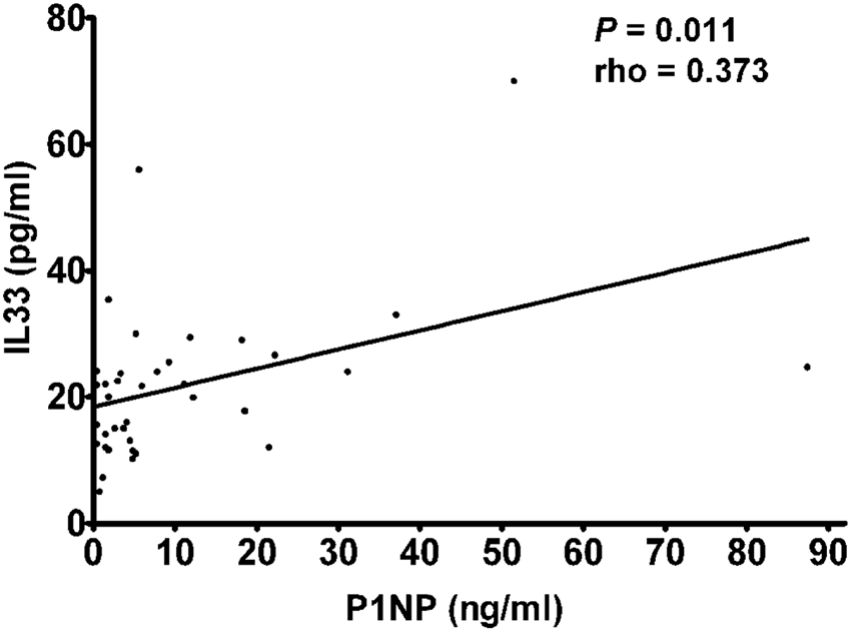
Correlation between IL-33 serum levels and N-terminal propeptide of type 1 procollagen (P1NP) in osteoporotic patients.

**Figure 4:**
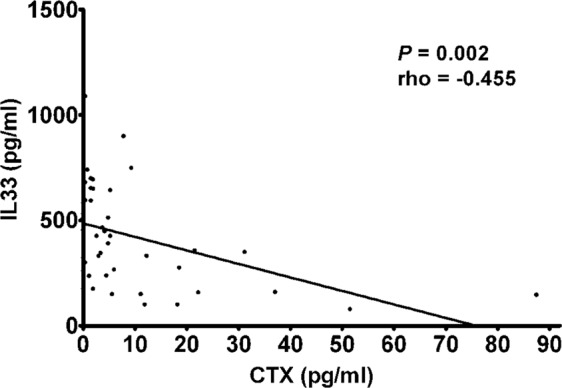
Correlation between IL-33 serum levels and C-terminal telopeptide of type 1 collagen (CTX) in osteoporotic patients.

There was a significant positive correlation in all patients with osteoporosis between serum levels of IL-33 and PTH (rho = 0.314; P = 0.026)(Fig. 2), as well as between serum levels of IL-33 and P1NP (rho = 0.373; P = 0.011) (Fig. 3), whereas a significant negative correlation was observed between serum levels of IL-33 and CTX (rho = −0.455; P = 0.002) (Fig. 4).

**Figure 5:**
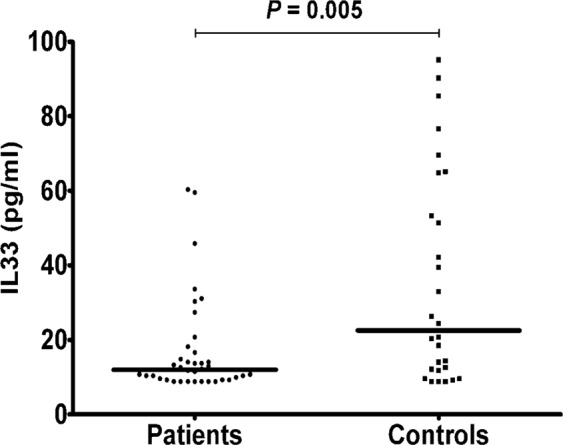
IL-33 serum levels in patients with mild osteoporosis (T-score > −3.5) and controls; lines represent medians.

Considering the severity of osteoporosis measured according to BMD values patients were divided into two groups: 12 patients (mean age 62.25 ± 12.29 years) with severe osteoporosis (T-score > −3.5) and 38 (mean age 66.42 ± 8.52 years) with mild osteoporosis (T-score > −3.5). Although higher in subjects with lower BMD, no statistically significant differences were detected between IL-33 levels in postmenopausal women with mild osteoporosis (3.16 ± 2.14 pg/ml) compared to those affected by more severe osteoporosis (8.35 ± 7.5 pg/ml), as well as between controls and patients with severe osteoporosis, whereas postmenopausal osteoporotic women exhibiting higher BMD showed significantly lower IL-33 levels compared to controls (P = 0.005) (Fig. 5).

**Figure 6:**
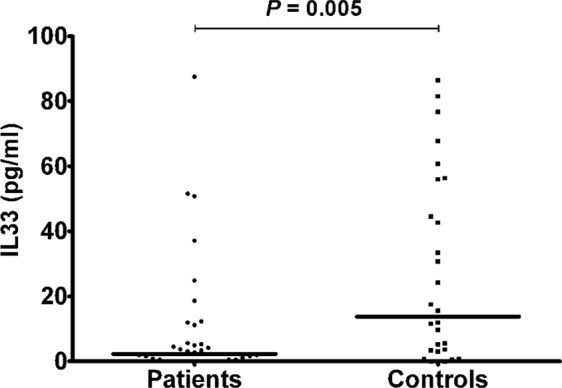
IL-33 serum levels in unfractured patients and controls; lines represent medians.

In addition, we divided patients, according to the presence or absence of osteoporotic fractures, into two groups: IL-33 serum levels, although tendentially higher in patients with fractures, was not significantly different between fractured (n. 14; mean age 65.29 ± 10.31 years) and unfractured (n. 36; mean age 65.47 ± 9.45 years) osteoporotic women (6.87 ± 3.52 pg/ml and 2.24 ± 3.13 pg/ml, respectively), neither between controls and fractured patients; significantly lower lL-33 levels were found in unfractured patients compare to controls (P = 0.005)(Fig. 6).

## Discussion

Although its original identification as an inducer of Th2 immune responses, IL-33 is now considered a pleiotropic cytokine: it can be released in extracellular space but can also act as an intracellular nuclear factor in inflammatory diseases and as an alarmin in response to cellular damage or mechanical injury^1^. It predominantly contributes to allergic airway inflammation^6^ and is protective against atherosclerosis^26^ and some types of infection^27^ but also plays a pivotal role in the development of many autoimmune diseases^5,7^. Therefore, depending on the disease, it can either promote the resolution of inflammation or drive disease pathology.

The effect of immune system and inflammation on bone is mediated by cytokines. However, not all cytokines involved in bone remodeling have an osteoclastogenic effect: some cytokines contribute to bone resorption, whereas other cytokines exert protective effects against osteoporosis, and others still have pleiotropic functions. The final effect of each of them is therefore dependent on the target cell and the combined action of the other cytokines and suppressor and/or stimulatory control factors involved in the various phases of the complex bone remodeling process that controls the health of our skeleton and ultimately the appearance and progression of osteoporosis^12^.

Our clinical results suggest that it is the anti-osteoclastic effect of IL-33 that is most critical in bone remodeling in postmenopausal women. Although the direct effect of IL-33 on osteoclast function or bone resorption is not clear, our results of a negative correlation between IL-33 and the resorption marker CTX seems to confirm that IL-33 inhibits osteoclast differentiation.

The lower levels of IL-33 which we found in osteoporotic patients are consistent with several observations that IL-33 inhibits osteoclast differentiation under experimental conditions^15,17,19^. In fact, IL-33 inhibits RANKL-dependent osteoclast formation, thus protecting from inflammatory bone loss^21,22^.

Furthermore, IL-33 also inhibits osteoclast differentiation by both inducing antiosteoclastogenic cytokines such as granulocyte-macrophage colony-stimulating factor (GM-CSF), IL-10, IL-4 and IFN-y and skewing the differentiation of osteoclast precursors toward alternatively activated macrophage and dendritic cells^15^.

Although it has been suggested a RANKL-like action of IL-33 in human osteoclast formation^28^, the IL-33 pro-resorptive effects on bone is weak, relative to the strong and consistent effects of RANKL, and highly variable, with some types of osteoclast progenitors able to differentiate into functional resorbing cells following IL-33 stimulation and other osteoclast progenitor-containing populations appearing unresponsive^29^. This could partially explain the apparently contrasting results of some *in vitro* studies which suggest a pro-resorptive and consequently pro-osteoporotic role of IL-33. Furthermore, the effects of IL-33 on bone may vary depending on the stage of the disease and hormonal influences and the final effect of IL-33 on bone is therefore strongly conditioned by the clinical context.

Moreover, the regulatory role of IL-33 in bone remodeling is also partially mediated by hormonal influences. In fact, IL-33 and its receptors are regulatory targets of PTH, notoriously a master factor in maintaining bone homeostasis and promoting bone formation when administered pharmacologically^17,30^. Not surprisingly, also IL-18, that is another member of the IL-1 family to which IL-33 belongs, is required for the bone-anabolic action of PTH^31^. A study by Saleh *et al*.^17^ showed that IL-33 mRNA levels in cultured calvarial osteoblasts were greatly enhanced by PTH and oncostatin M treatment. Our result of a positive correlation between PTH and IL-33 serum levels in osteoporotic postmenopausal women is in agreement with the observation that IL-33 is strongly stimulated by PTH, thus confirming that this cytokine may participate in the osteoblast-mediated anabolic effects of this hormone. This hypothesis is also supported by the finding of a positive correlation between IL-33 and the bone anabolic marker P1NP.

Generally, a Th1 profile and the hyperproduction of pro-inflammatory cytokines, mainly IL-1, IL-6, IL-17 and tumor necrosis factor (TNF)-α, condition the appearance and progression of both primitive and secondary osteoporosis. On the contrary, regulatory cytokines, such as IL-10, and Th2 cytokines, such as IL-4, exert a prevalent bone anabolic action so as to be regarded as protective against osteoporosis. Not surprisingly, IL-33, which potently enhances Th2 immune response, could exhibit a prevalent protective role in osteoporosis, which is considered a Th1/Th17 cell mediated inflammatory disease^1,32,33^. Our findings in osteoporotic patients are in agreement with this assumption, suggesting a prevalent protective role of this cytokine in postmenopausal osteoporosis.

On the other hand, recent observations both *in vitro* and *in vivo* have shown that inflammatory cytokines of the Th2 profile may also contribute to the development of osteoporosis. For example, in a recent study we have shown that the Th2 cytokine IL-31 was increased in postmenopausal women with decreased BMD, but this increase was not related with the severity of the disease^34^. In the present study on the contrary, serum levels of IL-33 are lower in postmenopausal osteoporosis compared to age-mached healthy women. However, as the disease progresses and the BMD decreases, the protective effect of IL-33 on bone tends to disappear, probably due to the interference of other factors and osteoclastogenic cytokines.

Therefore, IL-33 could be likely regarded as an important endogenous regulator of bone cell activity, mainly exerting antiosteoclastogenic and osteoblast stimulatory effects. However, since the balance between the various involved cytokines is very complex and plastic, IL-33 itself could also be a double-edged sword, protecting against osteoporosis through its biological action in bone metabolism, but also exerting a pro-resorptive role through its proinflammatory functions, depending on the specific clinical context and disease stage^35^.

Our results demonstrate that serum IL-33 levels are significantly lower in postmenopausal osteoporotic patients compared to controls. A positive correlation between IL-33 and PTH serum levels was also observed. To the best of our knowledge, this is the first published clinical report of both low IL-33 serum levels and positive correlation between IL-33 and PTH in postmenopausal osteoporotic women. Moreover, both the positive and negative correlation between IL-33 and bone formation and resorption markers respectively, indicate that this cytokine may exert a role in bone remodeling, likely influencing osteoblast and osteoclast function. These data suggest that IL-33 represents an important bone-protecting cytokine which may be a novel therapeutic tool in the prevention and therapy of osteoporosis in postmenopausal women and treatment with recombinant IL-33 could be useful for future strategies against osteoporosis.

## Data Availability

The complete collected data - clinical, anthropometric parameters, laboratory and instrumental tests- used to support the findings of this study are available from the corresponding author upon request.

## References

[1] Liew FY (2016). Interleukin-33 in health and disease. Nat Rev Immunol.

[2] Villarreal DO, Weiner DB (2014). Interleukin 33: a switch-hitting cytokine. Curr Opin Immunol.

[3] Molofsky AB, Savage A, Locksley RM (2015). Interleukin-33 in tissue homeostasis, injury and inflammation. Immunity.

[4] Demyanets, S. *et al*. Soluble ST2 and interleukin-33 levels in coronary artery disease: relation to disease activity and adverse outcome. *Plos One* **9**10.1371/journal.pone.0095055 (2014). 10.1371/journal.pone.0095055 PMC3994012 24751794

[5] Pei C (2014). Emerging role of interleukin-33 in autoimmune diseases. Immunology.

[6] Li R (2015). Interleukin-33 and receptor ST2 as indicators in patients with asthma: a meta-analysis. Int J Clin Exp Med.

[7] Xu WD (2013). IL-33 in rheumatoid arthritis: Potential role in pathogenesis and therapy. Hum Immunol.

[8] Xiangyang Z (2012). Increased levels of interleukin-33 associated with bone erosion and interstitial lung diseases in patients with rheumatoid arthritis. Cytokine.

[9] Miller, A. M. Role of IL-33 in inflammation and disease. *J Inflamm* **8**, (1), 10.1186/1476-9255-8-22 (2011). 10.1186/1476-9255-8-22 PMC3175149 21871091

[10] Takayanagi H (2015). Osteoimmunology in 2014: Two-faced immunology-from osteogenesis to bone resorption. Nat Rev Rheumatol.

[11] Zhao R (2012). Immune regulation of osteoclast function in postmenopausal osteoporosis: a critical interdisciplinary perspective. Int J Med Sci.

[12] Ginaldi L, De Martinis M (2016). Osteoimmunology and beyond. Curr Med Chem.

[13] Pacifici R (2016). T cells, osteoblasts, and osteocytes: interacting lineages key for the bone anabolic and catabolic activities of parathyroid hormone. Ann NY Acad Sci.

[14] Schulze J (2011). Interleukin-33 is expressed in differentiated osteoblasts and blocks osteoclast formation from bone marrow precursor cells. J Bone Min Res.

[15] Zaiss MM (2011). IL-33 shifts the balance from osteoclast to alternatively activated macrophage differentiation and protects from TNF-α-mediated bone loss. J Immunol.

[16] Cordero da Luz, F. A. *et al*. The physiopathological role of IL-33: new highlights in bone biology and a proposed role in periodontal disease. *Med Inflamm*, 10.1155/2014/342410 (2014). 10.1155/2014/342410 PMC3945897 24692848

[17] Saleh H (2011). Interleukin-33, a target of parathyroid hormone and oncostatin m, increases osteoblastic matrix mineral deposition and inhibits osteoclast formation *in vitro*. Endocrinology.

[18] Saidi S (2011). IL-33 is expressed in human osteoblasts, but has no direct effect on bone remodeling. Cytokine.

[19] Keller J (2012). Transgenic overexpression of interleukin-33 in osteoblasts results in decreased osteoclastogenesis. Biochem Biophys Res Commun.

[20] Mun SH (2010). Interleukin-33 stimulates formation of functional osteoclasts from human CD14+ monocytes. Cell Mol Life Sci.

[21] Kiyomiya H (2015). IL-33 inhibits RANKL-induced osteoclast formation through the regulation of Blimp-1 and IRF-8 expression. Biochem Biophys Res Commun.

[22] Zhu X (2017). Dectin-1 signaling inhibits osteoclastogenesis via IL-33-induced inhibition of NFATc1. Oncotarget.

[23] Velickovic M (2015). ST2 deletion increases inflammatory bone destruction in experimentally induced periapical lesions in mice. J Endod 2015.

[24] World Medical Association Declaration of Helsinki (2001). Ethical principles for medical research involving human subjects. Bull World Health Org.

[25] Kanis JA (2002). Diagnosis of osteoporosis and assessment of fracture risk. Lancet.

[26] López-Mejías, R. *et al*. Protective Role of the Interleukin 33 rs3939286 Gene Polymorphism in the Development of Subclinical Atherosclerosis in Rheumatoid Arthritis Patients. *Plos One*. **10**, 10.1371/journal.pone.0143153 (2015). 10.1371/journal.pone.0143153 PMC4646618 26571131

[27] Rostan O (2015). Crucial and Diverse Role of the Interleukin-33/ST2 Axis in Infectious Diseases. Infect Immun.

[28] Mine Y (2014). Involvement of ERK and p38 MAPK pathways on Interleukin-33-induced RANKL expression in osteoblastic cells. Cell Biol Int.

[29] Eeles DG (2015). Osteoclast formation elicited by interleukin-33 stimulation is dependent upon the type of osteoclast progenitor. Mol Cell Endocrinol.

[30] Cheloha RW (2015). PTH receptor-1 signalling - mechanistic insights and therapeutic prospects. Nat Rev Endocrinol.

[31] Raggatt LJ (2008). Interleukin-18 is regulated by parathyroid hormone and is required for its bone anabolic actions. J Biol Chem.

[32] Zhang J (2015). Changes of serum cytokines-related Th1/Th2/Th17 concentration in patients with postmenopausal osteoporosis. Gynecol Endocrinol.

[33] Saluja R (2015). The role of the IL-33/IL-1RL1 axis in mast cell and basophil activation in allergic disorders. Mol Immunol.

[34] Ginaldi L (2015). Increased levels of interleukin 31 (IL-31) in osteoporosis. BMC Immunol.

[35] Liew, F. Y. IL-33: a Janus cytokine. *Ann Rheum Dis* **71** (Suppl 2), 10.1136/annrheumdis-2011-200589 (2012). 10.1136/annrheumdis-2011-200589 22460136

